# Identification of molecular characteristics of hepatocellular carcinoma with microvascular invasion based on deep targeted sequencing

**DOI:** 10.1002/cam4.7043

**Published:** 2024-04-04

**Authors:** Linlin Zheng, Yaru Wang, Zhenrong Liu, Zhihao Wang, Changcheng Tao, Anke Wu, Haiyang Li, Ting Xiao, Zhuo Li, Weiqi Rong

**Affiliations:** ^1^ Department of Hepatobiliary Surgery, National Cancer Center/National Clinical Research Center for Cancer/Cancer Hospital Chinese Academy of Medical Sciences and Peking Union Medical College Beijing China; ^2^ State Key Laboratory of Molecular Oncology, Department of Etiology and Carcinogenesis, National Cancer Center/National Clinical Research Center for Cancer/Cancer Hospital Chinese Academy of Medical Sciences and Peking Union Medical College Beijing China; ^3^ Department of Hepatobiliary Hernia Surgery Liaocheng Dongcangfu People's Hospital Liaocheng China; ^4^ Department of Pathology, National Cancer Center/National Clinical Research Center for Cancer/Cancer Hospital Chinese Academy of Medical Sciences and Peking Union Medical College Beijing China

**Keywords:** deep targeted sequencing, hepatocellular carcinoma, microvascular invasion, molecular characteristics

## Abstract

**Background:**

As an indicator of tumor invasiveness, microvascular invasion (MVI) is a crucial risk factor for postoperative relapse, metastasis, and unfavorable prognosis in hepatocellular carcinoma (HCC). Nevertheless, the genetic mechanisms underlying MVI, particularly for Chinese patients, remain mostly uncharted.

**Methods:**

We applied deep targeted sequencing on 66 Chinese HCC samples. Focusing on the telomerase reverse transcriptase (TERT) promoter (TERTp) and TP53 co‐mutation (TERTp+/TP53+) group, gene set enrichment analysis (GSEA) was used to explore the potential molecular mechanisms of the TERTp+/TP53+ group on tumor progression and metastasis. Additionally, we evaluated the tumor immune microenvironment of the TERTp+/TP53+ group in HCC using multiplex immunofluorescence (mIF) staining.

**Results:**

Among the 66 HCC samples, the mutated genes that mostly appeared were TERT, TP53, and CTNNB1. Of note, we found 10 cases with TERTp+/TP53+, of which nine were MVI‐positive and one was MVI‐negative, and there was a co‐occurrence of TERTp and TP53 (*p* < 0.05). Survival analysis demonstrated that patients with the TERTp+/TP53+ group had lower the disease‐free survival (DFS) (*p* = 0.028). GSEA results indicated that telomere organization, telomere maintenance, DNA replication, positive regulation of cell cycle, and negative regulation of immune response were significantly enriched in the TERTp+/TP53+ group (all adjusted *p*‐values (*p*.adj) < 0.05). mIF revealed that the TERTp+/TP53+ group decreased CD8^+^ T cells infiltration (*p* = 0.25) and enhanced PDL1 expression (*p* = 0.55).

**Conclusions:**

TERTp+/TP53+ was significantly enriched in MVI‐positive patients, leading to poor prognosis for HCC patients by promoting proliferation of HCC cell and inhibiting infiltration of immune cell surrounding HCC. TERTp+/TP53+ can be utilized as a potential indicator for predicting MVI‐positive patients and poor prognosis, laying a preliminary foundation for further exploration of co‐mutation in HCC with MVI and clinical treatment.

## Graphical abstract

We applied deep targeted sequencing on 66 Chinese HCC samples and found that TERTp+/TP53+ could be used as a potential biomarker for predicting MVI‐positive patients and poor prognosis, laying a preliminary foundation for further exploration of co‐mutation in HCC with MVI and clinical treatment.

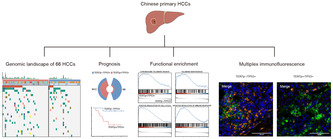

## 1 INTRODUCTION

Primary liver cancer (PLC) exhibits high heterogeneity and stands as one of the most prevalent malignant tumors of the digestive system globally.^1^ In China, PLC is the fourth most frequent malignant tumor and the second leading cause of cancer‐related mortality.^2^, ^3^ Hepatocellular carcinoma (HCC), the predominant type of PLC, is associated with several well‐known risk factors such as chronic viral hepatitis (HBV or HCV), excess alcohol consumption, aflatoxin B1 exposure, and metabolic diseases.^4^, ^5^, ^6^ Radical hepatectomy is still the most efficacious therapeutic approach for patients with early‐stage HCC. Unfortunately, only about 20% of patients can undergo surgery and face the problem of high recurrence and metastasis rates after surgery.^7^, ^8^

Microvascular invasion (MVI) is a well‐known risk factor for postoperative relapse and poor prognosis of HCC,^9^, ^10^ which symbolizes the present of tumor cell nest in vessels lined with the endothelium under the microscope, mainly in the portal vein.^11^ For patients undergoing hepatectomy or liver transplantation, MVI‐positive patients had a higher tumor recurrence rate.^12^, ^13^, ^14^ MVI can merely be identified by postoperative histopathological examination. Therefore, accurate preoperative prediction of MVI holds paramount importance in clinical decision, adjuvant therapy and prognosis evaluation of HCC patients.

Next‐generation sequencing technology enables us to identify the driver genes with oncogenic functions at the molecular level. Recent studies on comprehensive genomic profile of HCC have identified frequently mutated genes, including telomerase reverse transcriptase (TERT), TP53, and CTNNB1.^15^, ^16^, ^17^, ^18^ TERT, located at chromosome 5p15.33, serves as the catalytic subunit and rate‐limiting enzyme of telomerase, playing a crucial role in maintaining telomere length and controlling unlimited cellular proliferation.^19^, ^20^ Previous studies have indicated that hotspot mutations of TERT promoter (TERTp) (C228T and C250T) are detected in atypical hyperplastic nodules within cirrhotic livers. The mutation frequency significantly increases during the malignant transformation process: 6% in low‐grade dysplastic nodules (LGDN), 19% in high‐grade dysplastic nodules (HGDN), and 61% in early‐stage HCC.^21^ TERTp mutation is the earliest somatic mutation in the progression from atypical hyperplastic nodules to HCC.^22^, ^23^ TP53, located at chromosome 17p13.1, functions as a vital tumor suppressor and transcription factor. TP53 is activated after DNA damage, resulting in transcriptional upregulation of its target genes, thereby arresting the cell cycle, apoptosis, and DNA repair.^24^ However, the specific driver genes associated with MVI are still unclear, and the identification of driver genes can further understand the molecular mechanisms of HCC patients with MVI and provide potential therapeutic targets.

Among this study, we applied deep targeted sequencing to investigate somatic mutations in specific genes among 66 Chinese HCC patients with or without MVI. We found that the TERTp and TP53 co‐mutation (TERTp+/TP53+) group was significantly enriched in MVI‐positive patients, and there was a co‐occurrence relationship of TERTp and TP53 (*p* < 0.05). Consequently, we narrowed our focus to the TERTp+/TP53+ group to perform pathway enrichment analysis and used multiplex immunofluorescence (mIF) to evaluate the impact of co‐mutation on the tumor immune microenvironment (TIME).

## 2 METHODS

### 2.1 Study population

A total of 66 HCC samples were collected for deep targeted sequencing from January 2018 to October 2022 at National Cancer Center (NCC)/Cancer Hospital, Chinese Academy of Medical Sciences. Informed consent was obtained from all participants included in the study. This study was approved by the Ethics Committee of National Cancer Center/Cancer Hospital, Chinese Academy of Medical Sciences. We downloaded somatic mutation data of 365 HCC patients from The Cancer Genome Atlas (TCGA) database to identify mutation signatures and mutation landscape. The following cohort was obtained by screening samples with somatic mutation data, transcriptome data, and proteome data based on the Chinese population. A total of 110 HCC samples from Jiang et al (93 genome profiles, 35 transcriptional profiles, and 101 protein profiles)^25^ were utilized to research the relationship of mutation status with pathway enrichment and immune cell infiltration.

### 2.2 DNA extraction and capture‐based targeted DNA sequencing

We extracted DNA from 66 formalin‐fixed paraffin‐embedded (FFPE) samples using the QIAamp DNA FFPE Tissue Kit. Subsequently, DNA concentration was quantified by Qubit dsDNA assay. The A260/A280 values, ranging from 1.8 to 2.0, indicated high DNA purity. We employed a panel for deep targeted sequencing, which included 520 genes highly correlated with the development and progression of tumors. Selecting fragments ranging from 200 to 400 bp in size was performed using magnetic beads from Agencourt AMPure XP kit. Hybridization and incubation with biotin‐labeled capture probes, hybrid selection using magnetic beads, and PCR amplification were subsequently performed. Using the Bioanalyzer HS DNA assay for concentration and fragment size analysis of the purified library. Employing the Miseq instrument for paired‐end sequencing of samples that met the criteria.

### 2.3 Sequencing data analysis

We conducted quality control on the raw fastq data using the fastqc software, which involved trimming adapter sequences and removing low‐quality sequences. The fastq data was aligned to the human genome (hg19) using BWA 0.7.10.^26^ PCR duplicate reads were depleted prior to base substitution detection. GATK v3.2–2, MuTect and VarScan were employed for local alignment optimization and variant calling.^27^ SNVs and indels were identified and annotated using the dbNSFP (version 30a), COSMIC (version 69), and dbSNP (snp138) databases. Filtering out variants with mutation frequencies exceeding 1.0% in 1000 Genomes. The remaining mutations were annotated using ANNOVAR and SnpEff. DNA translocation research was performed via both Tophat2^28^ and Factera 1.4.3.^29^ The Integrative Genomics Viewer was employed to visually inspect the alignment of variants against the reference genome, ensuring the precision of variant calls.

### 2.4 Mutational signature analysis

To identify the mutation signatures in NCC HCC cohort and TCGA HCC cohort, the significance of gene mutations was determined by MutSig Covariate (MutSigCV) algorithm.^30^, ^31^ Based on the 96 mutation spectra (4 possible 5′ adjacent bases × 6 possible base substitution types (T > A, T > C, T > G, C > A, C > G, C > T) × 4 possible 3′ adjacent bases) in each tumor sample, with nTry set to six (maximum value), the nonnegative matrix factorization (NMF) method was employed to decompose point mutations into distinct mutation signatures. We utilized maftools::compareSignatures to compare the identified mutation signatures with the 30 known mutation signatures in the COSMIC database, calculating cosine similarity to determine the best match.

### 2.5 Gene set enrichment analysis (GSEA)

To explore the potential molecular mechanisms underlying the TERTp+/TP53+ group, based on the transcriptome data, we used the R package clusterProfiler to perform GSEA between the TERTp+/TP53+ group and the TERTp wild‐type and TP53 mutation (TERTp‐/TP53+) group. Pathways with normalized enrichment score (NES) >1, adjusted *p*‐value (*p*.adj) <0.05 and false discovery rate (fdr) <0.25 were considered statistically significantly enriched.

### 2.6 CIBERSORT analysis

To evaluate the impact of mutation status on the TIME, based on the deconvolution algorithm CIBERSORT,^32^ we applied transcriptome data and proteome data to assess the relative proportions of 22 immune cells in the TERTp+/TP53+ and the TERTp‐/TP53+ groups.

### 2.7 Multiple immunofluorescence (mIF) staining

Different primary antibodies were used to analyze the immune microenvironment, containing CD8 antibody (clone C8/144B, CST70306, CST) and PDL1 antibody (clone E1L3N, CST13684, CST). The diluted primary antibody solution was dropped onto the tissue sections and incubated at room temperature for 1 h. The secondary antibody from the kit was added dropfold and incubated for 10 min at room temperature in the dark. Selecting suitable dye channels, the tyramide signal amplification signal amplification solution was diluted at a ratio of 1:100. Cell nuclei were counterstained with DAPI. Imaging of the slides was performed using the Mantra System (Perkin Elmer), with a 20 nm wavelength interval, for broad‐spectrum fluorescence imaging in the range of 420–720 nm.

### 2.8 Image analysis

Extracting spectra of each fluorophore and tissue autofluorescence from images of unstained and single‐stained tissues, respectively, to construct the spectral library necessary for multispectral unmixing. The analysis was performed using Inform software. With the assistance of the spectral library, we generated reconstructed images of the sections, effectively eliminating autofluorescence. In this study, we assessed the density and positive rate of CD8 and PDL1 staining on lymphocytes (LYMs) within the parenchyma of the tissue samples.

### 2.9 Statistical analysis

R 4.2.2 software was performed for statistical analysis of bioinformatics data in this study. For continuous data, appropriate statistical tests such as the Student's *t*‐test or the nonparametric Mann–Whitney test were applied. Categorical variables were compared using chi‐squared test or Fisher's exact test. Disease‐free survival (DFS) was defined as the period starting from the initiation of therapy and ending at the occurrence of disease recurrence or death resulting from disease progression. *p* < 0.05 was considered statistically significant.

## 3 RESULTS

### 3.1 Mutation signatures of the Chinese HCC patients

**FIGURE 1:**
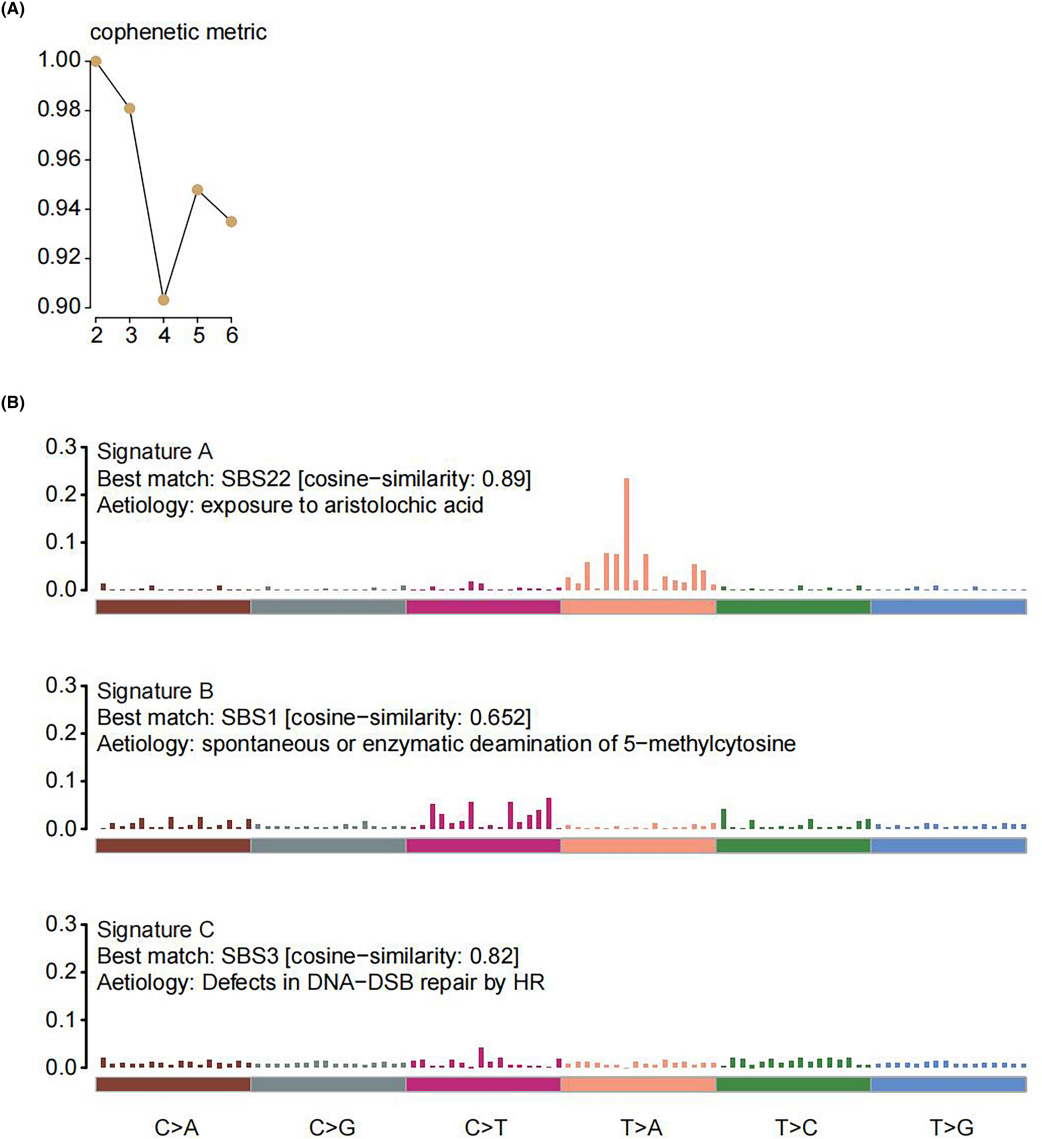
The identification of mutation signatures in 66 hepatocellular carcinoma (HCC) patients in national cancer center (NCC) cohort. (A) Nonnegative matrix factorization (NMF). (B) Patterns of three mutation signatures (signatures A‐C) identified in 66 HCC patients. HCC, hepatocellular carcinoma; NCC, national cancer center; NMF, nonnegative matrix factorization.

**FIGURE 2:**
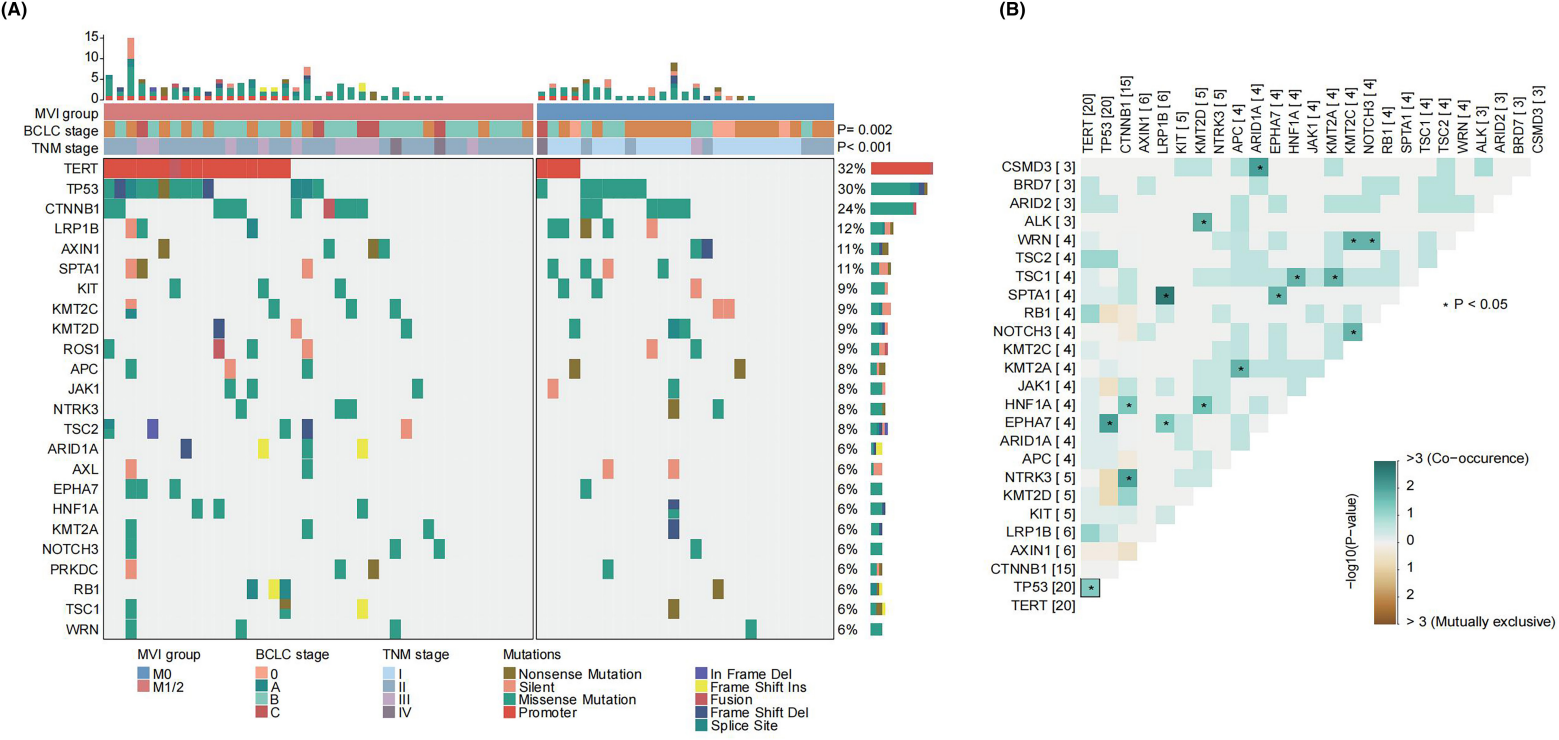
Genomic landscape in hepatocellular carcinoma (HCC) patients with or without microvascular invasion (MVI) in national cancer center (NCC) cohort. (A) Genetic profile and the associated clinicopathologic features of 66 HCC patients. (B) Co‐occurrence and mutually exclusive of mutated genes in 66 HCC patients. Green represents the co‐occurrence mutations; yellow represents the mutual exclusive mutations. HCC, hepatocellular carcinoma; MVI, microvascular invasion; NCC, national cancer center. *p < 0.05.

To understand the genomic alterations in Chinese HCC patients, we performed comprehensive somatic mutation analysis on deep targeted sequencing data from 66 HCC patients. According to the cophenetic curve of NMF method, the optimal cluster number was three (Figure 1A). Mutation signatures of 96 mutation spectra identified three signatures in 66 HCC patients (Figure 1B). Signature A exhibited highly similarity to COSMIC signature 22 (cosine similarity = 0.89) and was associated with exposure to aristolochic acid. Signature B was similar to COSMIC signature 1 (cosine similarity = 0.652) and was associated with spontaneous or enzymatic deamination of 5‐methylcytosine. Signature C exhibited highly similarity to COSMIC signature 3 (cosine similarity = 0.82) and was associated with defects in DNA‐DSB repair by HR. We compared the mutation signatures between Chinese HCC patients (signatures A‐C) and the TCGA HCC cohort (signatures a‐d), revealing a similarity in the proposed etiology of signature C (COSMIC signature 3) and signature c (COSMIC signature 6), both involving defective DNA repair. The commonality lay in the identification of COSMIC signature 22 (aristolochic acid) by both signature A and signature d (Figure 1B, Figure S1A). The proportion of COSMIC signature 22 (aristolochic acid) in the TCGA HCC cohort was found to be very low (25/365, 6.8%) (Figure S1B). Consistently, COSMIC signature 22 (aristolochic acid) was more prevalent among HCC patients in China compared to Western countries. In addition, compared to the somatic mutation landscape from the TCGA HCC cohort, Chinese HCC patients exhibited a higher proportion of TERT mutations (32% [21/66] vs. 0.5% [2/365]), while the proportion of TP53 mutations was similar (30% [20/66] vs. 30% [110/365]) (Figure 2A, Figure S1C). Considering the etiology of HCC, HBV infection was the major risk factor for HCC in China, HCV infection and excess alcohol consumption were the major risk factors for HCC in Western countries. Disparities in populations and etiologies contributed to differences in the associated driver genes, mutation frequencies, and mutation signatures.

### 3.2 Clinical characteristics and molecular mutations of HCC patients with or without MVI

**FIGURE 3:**
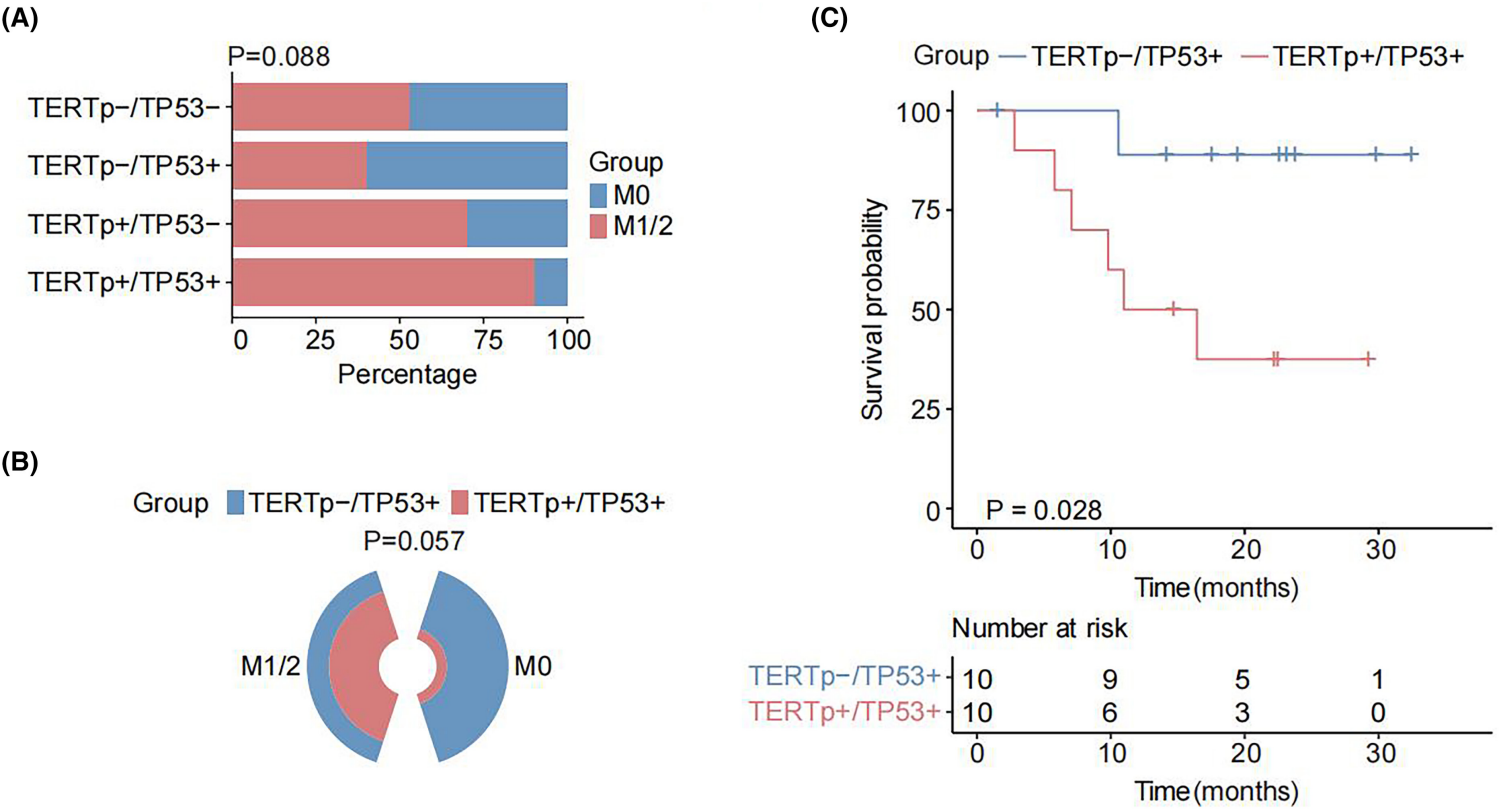
Association between TERTp and TP53 mutation and microvascular invasion (MVI) in national cancer center (NCC) hepatocellular carcinoma (HCC) cohort. (A, B) Correlation between mutation status and MVI. (C) Survival analysis showed that the TERTp+/TP53+ group (*n* = 10) had significantly shorter disease‐free survival (DFS). TERTp, telomerase reverse transcriptase promoter; MVI, microvascular invasion; NCC, national cancer center; HCC, hepatocellular carcinoma; TERTp+/TP53+, TERT promoter (TERTp) and TP53 co‐mutation; DFS, disease‐free survival.

Based on postoperative pathological results, 66 HCC patients were categorized into the MVI‐positive group (39 cases) and MVI‐negative group (27 cases). We found that compared to the MVI‐negative group, the MVI‐positive group exhibited a significantly higher proportion of patients with Barcelona clinic liver cancer (BCLC) stage B‐C, tumor node metastasis stage II‐IV, maximum diameter of the primary tumor >5 cm, and hepatic capsule invasion (Table 1, all *p* < 0.05). Using next‐generation sequencing results from 66 HCC samples, we compared the somatic mutation frequencies in specific genes between the MVI‐positive and MVI‐negative groups. The common mutated genes were TERT, TP53, and CTNNB1 (Figure 2A). Among the driver genes of HCC, TERT was the most common gene alterations, of which 20 mutation types were promoter mutations and 1 mutation type was a fusion mutation. Therefore, our subsequent analyses focused on TERTp. It was worth noting that we found nine cases of TERTp+/TP53+ in the MVI‐positive group and only one case of TERTp+/TP53+ in the MVI‐negative group. Furthermore, we calculated the co‐occurrence and mutually exclusive of the most common mutated genes and found a co‐occurrence relationship between TERTp and TP53 (Figure 2B, *p* < 0.05). Further analysis of the correlation between mutation status and MVI indicated that the TERTp+/TP53+ group was significantly enriched in MVI‐positive group, and the TERTp‐/TP53+ group was significantly enriched in MVI‐negative group (Figure 3A, *p* = 0.088; Figure 3B, *p* = 0.057). Thus, we categorized the cohort into two groups: the TERTp+/TP53+ group and the TERTp‐/TP53+ group, and analyzed the relationship between the two groups and clinicopathological characteristics of HCC patients (Table 2). Patients in the TERTp+/TP53+ group were correlated with lower alpha fetoprotein (AFP) level (*p* = 0.020) and MVI‐positive (*p* = 0.057). More importantly, survival analysis demonstrated that the TERTp+/TP53+ group had significantly shorter DFS and the TERTp‐/TP53+ group had significantly longer DFS in NCC HCC cohort (Figure 3C, *p* = 0.028). Our findings suggested that TERTp+/TP53+ defined a distinct subgroup of MVI‐positive patients and poor prognosis.

**TABLE 1:** Clinicopathological characteristics of hepatocellular carcinoma (HCC) patients with or without microvascular invasion (MVI) in national cancer center (NCC) cohort.

Characteristic	M0	M1/2	*p*‐value
*n*	27	39	
Age, *n* (%)			
≤60	19 (28.8%)	26 (39.4%)	0.961
>60	8 (12.1%)	13 (19.7%)	
Gender, *n* (%)			
Female	3 (4.5%)	2 (3%)	0.393
Male	24 (36.4%)	37 (56.1%)	
Hypertension, *n* (%)			
No	24 (36.4%)	24 (36.4%)	0.030
Yes	3 (4.5%)	15 (22.7%)	
Portal hypertension, *n* (%)			
No	17 (25.8%)	32 (48.5%)	0.145
Yes	10 (15.2%)	7 (10.6%)	
Preoperative ALT(U/L), *n* (%)			
≤40	15 (22.7%)	26 (39.4%)	0.511
>40	12 (18.2%)	13 (19.7%)	
Preoperative AST (U/L), *n* (%)			
≤40	21 (31.8%)	29 (43.9%)	0.979
>40	6 (9.1%)	10 (15.2%)	
Preoperative AFP, *n* (%)			
≤200	14 (21.2%)	25 (37.9%)	0.459
>200	13 (19.7%)	14 (21.2%)	
Preoperative HBsAg, *n* (%)			
Negative	5 (7.6%)	11 (16.7%)	0.541
Positive	22 (33.3%)	28 (42.4%)	
Macrovascular invasion, *n* (%)			
No	27 (40.9%)	37 (56.1%)	0.509
Yes	0 (0%)	2 (3%)	
Hepatic capsule invasion, *n* (%)			
No	24 (36.4%)	15 (22.7%)	<0.001
Yes	3 (4.5%)	24 (36.4%)	
Number of tumors, *n* (%)			
1	20 (30.3%)	32 (48.5%)	0.636
≥2	7 (10.6%)	7 (10.6%)	
Maximum diameter of primary tumor (cm), *n* (%)			
≤5	23 (34.8%)	22 (33.3%)	0.028
>5	4 (6.1%)	17 (25.8%)	
BCLC stage, *n* (%)			
0	4 (6.1%)	0 (0%)	0.002
A	15 (22.7%)	11 (16.7%)	
B	7 (10.6%)	23 (34.8%)	
C	1 (1.5%)	5 (7.6%)	
TNM stage, *n* (%)			
I	19 (28.8%)	0 (0%)	< 0.001
II	6 (9.1%)	27 (40.9%)	
III	1 (1.5%)	10 (15.2%)	
IV	1 (1.5%)	2 (3%)	
Edmondson‐Steiner classification, *n* (%)			
I‐II	16 (24.6%)	15 (23.1%)	0.186
III‐IV	11 (16.9%)	23 (35.4%)	
Preoperative LYM, median (IQR)	1.81 (1.64, 3.11)	1.7 (1.34, 1.93)	0.035

Abbreviations: AFP, alpha fetoprotein; ALT, alanine transaminase; AST, aspartate aminotransferase; BCLC, Barcelona clinic liver cancer; HBsAg, hepatitis B surface antigen; HCC, hepatocellular carcinoma; LYM, lymphocyte.; MVI, microvascular invasion; NCC, national cancer center; TNM, tumor node metastasis.

**TABLE 2:** Clinicopathological characteristics of the TERTp+/TP53+ and the TERTp‐/TP53+ groups in national cancer center (NCC) hepatocellular carcinoma (HCC) cohort.

Characteristic	TERTp‐/TP53+	TERTp+/TP53+	*p*‐value
*n*	10	10	
Age, *n* (%)			
≤60	8 (53.3%)	7 (46.7%)	1.000
>60	2 (40%)	3 (60%)	
Gender, *n* (%)			
Female	2 (100%)	0 (0%)	0.474
Male	8 (44.4%)	10 (55.6%)	
Hypertension, *n* (%)			
No	6 (54.5%)	5 (45.5%)	1.000
Yes	4 (44.4%)	5 (55.6%)	
Portal hypertension, *n* (%)			
No	6 (42.9%)	8 (57.1%)	0.628
Yes	4 (66.7%)	2 (33.3%)	
Preoperative ALT (U/L), *n* (%)			
≤40	7 (46.7%)	8 (53.3%)	1.000
>40	3 (60%)	2 (40%)	
Preoperative AST (U/L), *n* (%)			
≤40	9 (60%)	6 (40%)	0.303
>40	1 (20%)	4 (80%)	
Preoperative AFP, *n* (%)			
≤200	3 (25%)	9 (75%)	0.020
>200	7 (87.5%)	1 (12.5%)	
Preoperative HBsAg, *n* (%)			
Negative	1 (16.7%)	5 (83.3%)	0.141
Positive	9 (64.3%)	5 (35.7%)	
Preoperative HBeAg, *n* (%)			
Negative	8 (47.1%)	9 (52.9%)	1.000
Positive	2 (66.7%)	1 (33.3%)	
Microvascular invasion, *n* (%)			
M0	6 (85.7%)	1 (14.3%)	0.057
M1/2	4 (30.8%)	9 (69.2%)	
Macrovascular invasion, *n* (%)			
No	10 (52.6%)	9 (47.4%)	1.000
Yes	0 (0%)	1 (100%)	
Hepatic capsule invasion, *n* (%)			
No	6 (60%)	4 (40%)	0.656
Yes	4 (40%)	6 (60%)	
Number of tumors, *n* (%)			
1	7 (46.7%)	8 (53.3%)	1.000
≥2	3 (60%)	2 (40%)	
Maximum diameter of primary tumor (cm), *n* (%)			
≤5	7 (46.7%)	8 (53.3%)	1.000
>5	3 (60%)	2 (40%)	
BCLC stage, *n* (%)			
A	4 (44.4%)	5 (55.6%)	0.714
B	5 (62.5%)	3 (37.5%)	
C	1 (33.3%)	2 (66.7%)	
TNM stage, *n* (%)			
I	4 (100%)	0 (0%)	0.118
II	5 (41.7%)	7 (58.3%)	
III	1 (33.3%)	2 (66.7%)	
IV	0 (0%)	1 (100%)	
Edmondson‐Steiner classification, *n* (%)			
I‐II	4 (57.1%)	3 (42.9%)	1.000
III‐IV	6 (46.2%)	7 (53.8%)	

Abbreviations: AFP, alpha fetoprotein; ALT, alanine transaminase; AST, aspartate aminotransferase; BCLC, Barcelona clinic liver cancer; HBsAg, hepatitis B surface antigen; LYM, lymphocyte; NCC, national cancer center; TERTp‐/TP53+, TERT promoter (TERTp) wild‐type and TP53 mutation; TERTp+/TP53+, TERT promoter (TERTp) and TP53 co‐mutation; TNM, tumor node metastasis.

### 3.3 Pathway enrichment analysis of TERTp and TP53 co‐mutation

**FIGURE 4:**
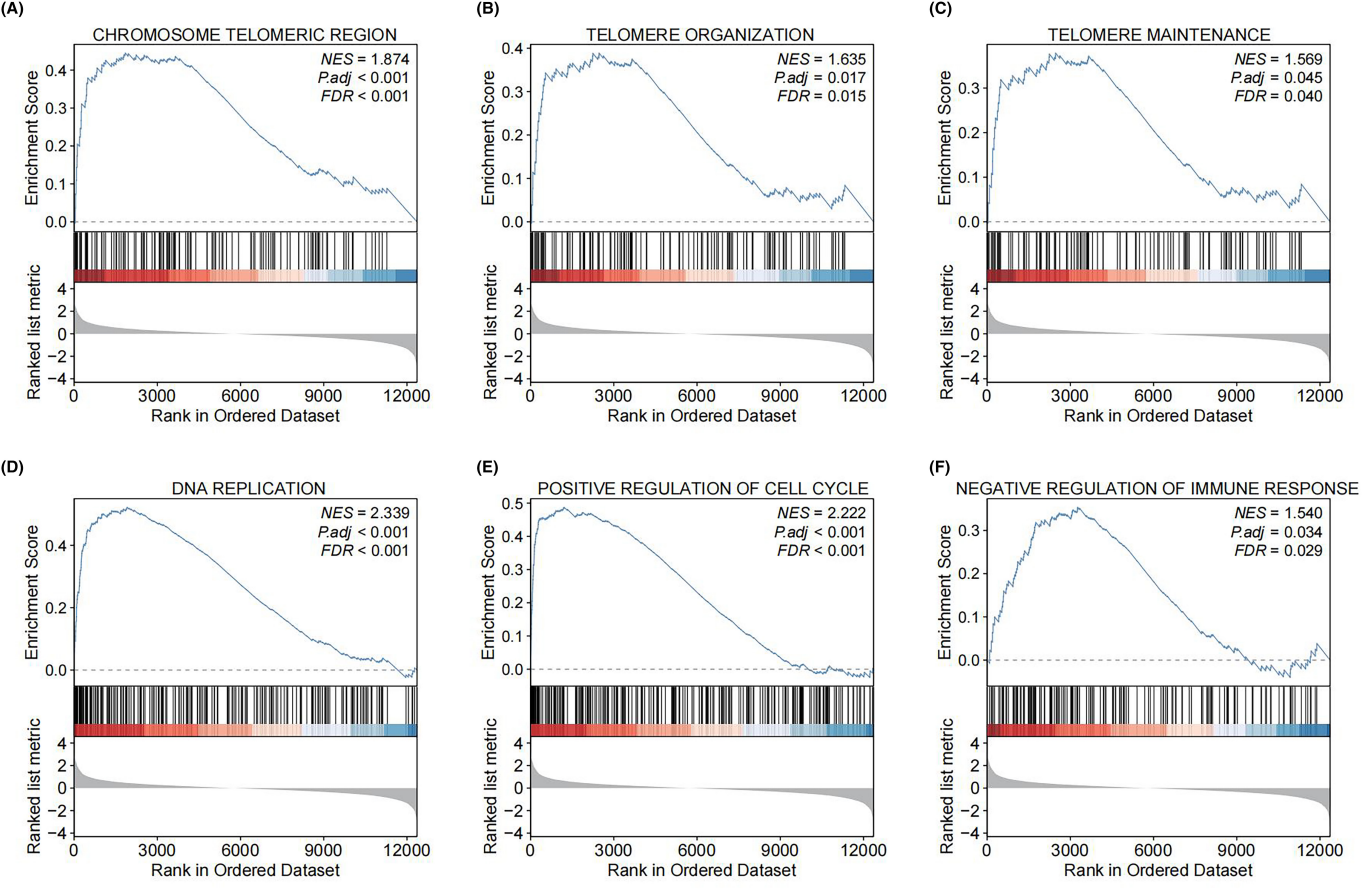
Pathway enrichment analysis between the TERTp+/TP53+ group and the TERTp‐/TP53+ group based on transcriptome data. (A) Chromosome telomeric region. (B) Telomere organization. (C) Telomere maintenance. (D) DNA replication. (E) Positive regulation of cell cycle. (F) Negative regulation of immune response. TERTp+/TP53+, TERT promoter (TERTp) and TP53 co‐mutation; TERTp‐/TP53+, TERT promoter (TERTp) wild‐type and TP53 mutation.

GSEA was performed to further explore the biological difference between the TERTp+/TP53+ group and the TERTp‐/TP53+ group. The results indicated that chromosome telomeric region, telomere organization, telomere maintenance, DNA replication, positive regulation of cell cycle, and negative regulation of immune response were significantly enriched in the TERTp+/TP53+ group (Figure 4, all *p*.adj < 0.05). The above findings suggested that the potential mechanisms of the TERTp+/TP53+ group in the occurrence and development of HCC, offering valuable insights for further research.

### 3.4 Immune cell infiltration of TERTp and TP53 co‐mutation

**FIGURE 5:**
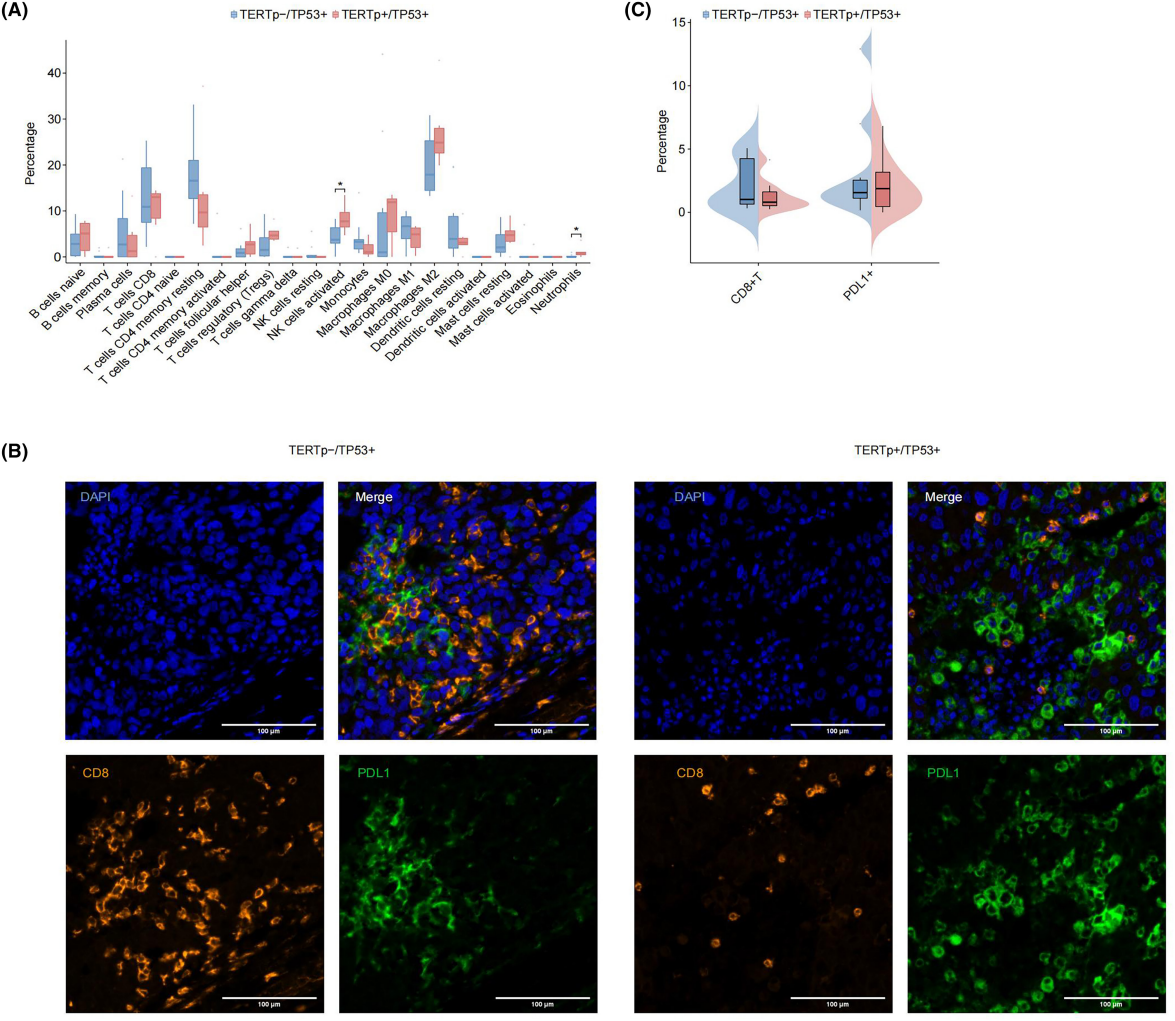
Immune cell infiltration landscapes based on TERTp and TP53 co‐mutation. (A) CIBERSORT immune infiltration analysis between the TERTp+/TP53+ group and the TERTp‐/TP53+ group based on transcriptome data. (B) Representative image of multiple immunofluorescence staining with CD8 (orange), PDL1 (green), and DAPI (blue) at scale bar 100 μm in national cancer center (NCC) hepatocellular carcinoma (HCC) cohort. (C) Detection of CD8 and PDL1 expression by multiple immunofluorescence (mIF) staining in the tumor parenchyma in NCC HCC cohort. TERTp, telomerase reverse transcriptase promoter; TERTp+/TP53+, TERT promoter (TERTp) and TP53 co‐mutation; TERTp‐/TP53+, TERT promoter (TERTp) wild‐type and TP53 mutation; HCC, hepatocellular carcinoma; mIF, multiple immunofluorescence; NCC, national cancer center. *p < 0.05.

The CIBERSORT was employed to calculate the infiltration degree of 22 immune cells between the two groups. Based on the transcriptome data, the TERTp+/TP53+ group displayed higher infiltration levels of NK cells activated and neutrophils compared to the TERTp‐/TP53+ group (Figure 5A, both *p* < 0.05). Based on proteome data, the TERTp+/TP53+ group had higher infiltration of NK cells resting and lower infiltration of T cells CD4 memory activated (Figure S2, both *p* < 0.05). mIF results revealed that the TERTp+/TP53+ group had lower infiltration of CD8^+^ T cells (*p* = 0.25) and higher PDL1 expression (*p* = 0.55) in the tumor parenchyma (Figure 5B,C). In general, the TERTp+/TP53+ group inhibited the immune effector cells in the tumor microenvironment.

## 4 DISCUSSION

HCC is a multistep‐process resulting from the accumulative of genetic mutations. This study delineated the genetic events that characterize HCC in China. The mutational signature analysis of deep targeted sequencing data from 66 HCC samples revealed three mutation signatures. Among them, signature A was found to be highly similar to COSMIC signature 22 that had been depicted and validated in previous studies (featured by dominant T > A mutation), which was a typical mutation of HCC relevant to aristolochic acid.^33^ In addition, consistent with the previous results, COSMIC signature 22 (aristolochic acid) was more prevalent in China than in Western countries.

MVI is an important risk factor for postoperative relapse of HCC and can merely be verified by postoperative pathological examination.^34^ In this study, we performed an integrative study of the somatic mutation profile of HCC patients with or without MVI. TERT, TP53, and CTNNB1 were the most frequently mutated driver genes. It was worth noting that TERTp+/TP53+ was found in 10 patients, of which nine were MVI‐positive and one was MVI‐negative, and there was a co‐occurrence relationship between TERTp and TP53. Further analysis of the correlation between mutation status and MVI indicated that the TERTp+/TP53+ group was significantly enriched in MVI‐positive patients, and the TERTp‐/TP53+ group was significantly enriched in MVI‐negative patients. More importantly, the TERTp+/TP53+ group had significantly shorter DFS and the TERTp‐/TP53+ group had significantly longer DFS (*p* = 0.028). Additionally, we performed pathway enrichment and immune infiltration analysis on the two groups to uncover the effect of co‐mutation on tumorigenesis and the degree of LYM infiltration in HCC.

TERT, as the catalytic subunit of telomerase, is essential for telomerase activity. TERTp creates binding sites for ETS‐family transcription factors to increase TERT expression.^35^, ^36^ The upregulation of TERT expression and resulting telomerase activity appears in the majority of human malignancies and are related to tumor progression and aggressiveness, including HCC, melanoma, urothelial carcinoma, glioma, and thyroid cancer.^37^, ^38^, ^39^, ^40^, ^41^ Ningarhari et al. found that the TERT overexpression group was significantly enriched to pathways such as telomere elongation and telomere maintenance and was associated with high tumor proliferation and poor prognosis.^42^ TP53 regulates various genes involved in cell cycle arrest, apoptosis, senescence, and DNA damage repair.^43^ In at least half of HCC patients with R249S TP53 mutation (12%–48%), alterations in the P53 cell cycle signaling pathway have been observed.^44^ Mutant TP53 causes dysregulation of the cell cycle, which allows replication of damaged DNA, ultimately leading to uncontrolled cell proliferation and tumorigenesis.^45^ However, the mechanisms of action of the TERTp+/TP53+ group in HCC remains unknown. Interestingly, according to our GSEA results, multiple signaling pathways (telomere organization, telomere maintenance, DNA replication, positive regulation of cell cycle, and negative regulation of immune response) were enriched in the TERTp+/TP53+ group (all *p*.adj < 0.05). The results indicated that the TERTp+/TP53+ group was not only enriched in cancer‐related pathways, such as telomere maintenance and the cell cycle, but also involved immune‐related pathways.

The tumor microenvironment is composed of tumor cells, immune cells, endothelial cells, fibroblasts, and cytokines. HCC is a cancer that occurs under the background of immunosuppression, involving decreased immune recognition or increased resistance to immune attacks, promoting tumor growth. We used the CIBERSORT algorithm to evaluate the relative abundance of 22 immune cells between the two groups. Based on the transcriptome data, the TERTp+/TP53+ group displayed higher infiltration levels of NK cells activated and neutrophils (both *p* < 0.05). Based on proteome data, the TERTp+/TP53+ group had higher infiltration of NK cells resting and lower infiltration of T cells CD4 memory activated (both *p* < 0.05). mIF was used to further explore the characteristics of TIME between the two groups. We found lower CD8^+^ T cells infiltration (*p* = 0.25) and elevated PDL1 expression (*p* = 0.55) in the tumor parenchyma of the TERTp+/TP53+ group. CD8^+^ T cells kill tumor cells by releasing granules containing perforin and granzyme or by cytokines such as TNF‐alpha and IFN‐gamma.^46^ Gabrielson et al. found that high CD8^+^ T‐cell density was significantly associated with lower relapse rates and longer recurrence free survival. PDL1 expressed on the surface of tumor cells can bind to PD1 on the surface of T cells, thereby inhibiting T cell proliferation and cytokine secretion, leading to T cell apoptosis and exhaustion. Increasing evidences have demonstrated that PD‐L1 overexpression is associated with poor prognosis in a variety of human cancers, including melanoma, lung cancer and HCC.^47^, ^48^, ^49^, ^50^ Decreased infiltration of CD8^+^ T cells and higher PDL1 expression suggested that TERTp+/TP53+ group may inhibit anti‐tumor immunity and promote the occurrence and development of HCC. Our study also has some limitations. The conclusions obtained require confirmed by a large‐sample prospective analysis.

## 5 CONCLUSION

TERTp+/TP53+ was a promising novel biomarker for predicting MVI‐positive patients and poor prognosis, which may guide treatment and relapse surveillance in HCC patients.

## AUTHOR CONTRIBUTIONS

**Linlin Zheng:** Formal analysis (lead); writing – original draft (lead). **Yaru Wang:** Investigation (equal); software (equal). **Zhenrong Liu:** Investigation (equal); software (equal). **Zhihao Wang:** Investigation (equal); software (equal). **Changcheng Tao:** Investigation (equal); software (equal). **Anke Wu:** Investigation (equal); software (equal). **Haiyang Li:** Investigation (equal); software (equal). **Ting Xiao:** Project administration (equal); supervision (equal); writing – review and editing (equal). **Zhuo Li:** Project administration (equal); supervision (equal); writing – review and editing (equal). **Weiqi Rong:** Funding acquisition (lead); project administration (equal); supervision (equal); writing – review and editing (equal).

## CONFLICT OF INTEREST STATEMENT

The authors declare no conflicts of interest.

## ETHICS STATEMENT

Informed consent was obtained from all participants included in the study. This study was approved by the Ethics Committee of National Cancer Center (NCC)/Cancer Hospital, Chinese Academy of Medical Sciences.

## Supporting information


Figure S1.

Figure S2.

## Data Availability

All the data are available from the corresponding author on reasonable request.
