# Supplementary figures and images for: Identification of molecular characteristics of hepatocellular carcinoma with microvascular invasion based on deep targeted sequencing
Source: Cancer Med. 2024 Apr 4;13(7):e7043. doi: 10.1002/cam4.7043 (PMC10993708; doi:10.1002/cam4.7043)

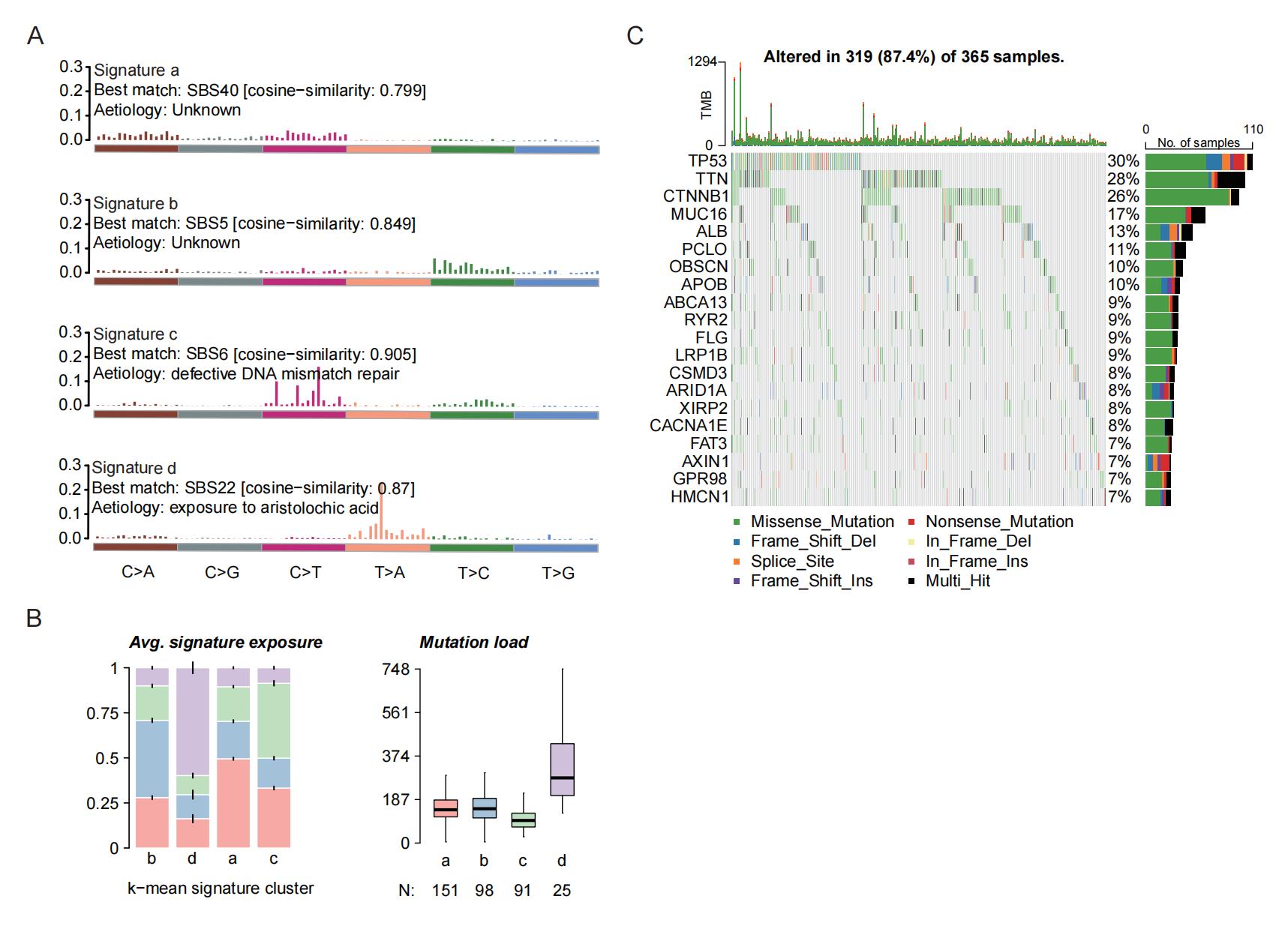

Supplement: Supplementary file 1 — Figure S1. [file CAM4-13-e7043-s002.jpg]

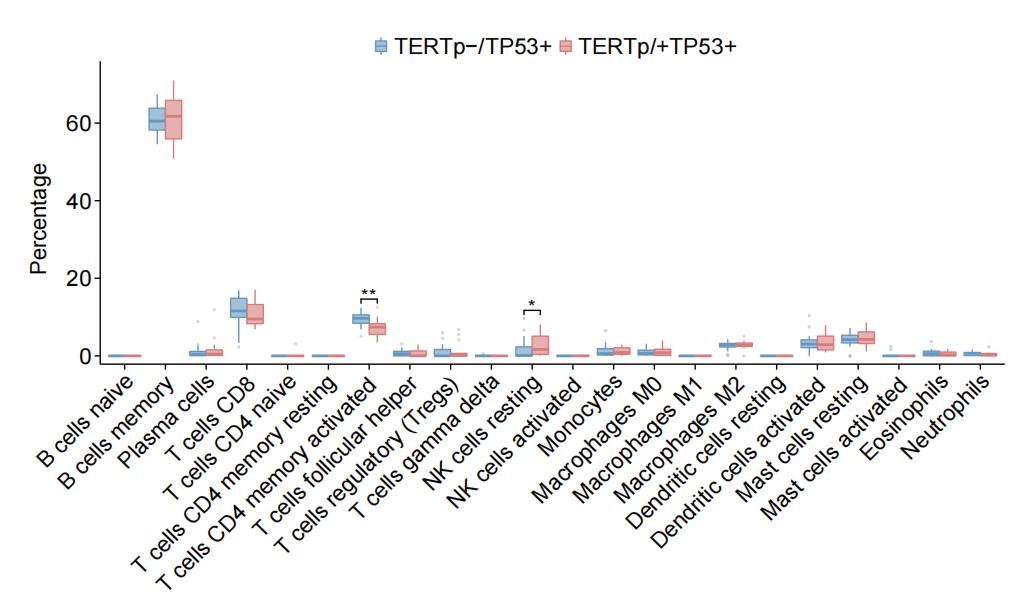

Supplement: Supplementary file 2 — Figure S2. [file CAM4-13-e7043-s001.jpg]
